# Supplementary material for: Asynchronous magnetic resonance elastography: Shear wave speed reconstruction using noise correlation of incoherent waves
Source: Magn Reson Med. 2022 Oct 27;89(3):990–1001. doi: 10.1002/mrm.29502 (PMC9792433; doi:10.1002/mrm.29502)
Supplement: Supplementary file 1 — DATA S1 MATLAB code used to generate the numerical simulations of Figure 1 [file MRM-89-990-s002.zip › k-Wave/helpfiles/waterAbsorption.html]

waterAbsorption :: Functions (k-Wave)


# waterAbsorption

Calculate ultrasound absorption in distilled water.

## Syntax

```
abs = waterAbsorption(f, T)
```

## Description

`waterAbsorption` calculates the ultrasonic absorption in distilled water at a given temperature and frequency using a 7th order polynomial fitted to the data given by Pinkerton (1949, The Absorption of Ultrasonic Waves in Liquids and its Relation to Molecular Constitution, Proceedings of the Physical Society. Section B, 2, 129-141). A plot of the absorption at 20degC returned by `waterAbsorption` along with the absorption calculated by the corresponding power law given by Szabo (2004, Diagnostic Ultrasound Imaging) is given below.

```
% calculate absorption between 0 and 50 MHz
f = 0:2:50;                     % [MHz]
abs = waterAbsorption(f, 20);  % [dB/cm]

% plot absorption and power law
figure;
plot(f, abs, 'k-', f, 2.17e-3 * f.^2, 'bx');
xlabel('Frequency [MHz]');
ylabel('Absorption Coefficient [dB/cm]');
```

## Inputs

|  |  |
| --- | --- |
| `f` | array of frequency values [MHz] |
| `T` | array of water temperature values [degC] |

## Outputs

|  |  |
| --- | --- |
| `abs` | absorption [dB/cm] |

## See Also

`waterDensity`, `waterNonlinearity`, `waterSoundSpeed`
